# The Dual Role of the Liver in Nanomedicine as an Actor in the Elimination of Nanostructures or a Therapeutic Target

**DOI:** 10.1155/2020/4638192

**Published:** 2020-02-24

**Authors:** Lorena Baboci, Sara Capolla, Federica Di Cintio, Federico Colombo, Prisca Mauro, Michele Dal Bo, Monica Argenziano, Roberta Cavalli, Giuseppe Toffoli, Paolo Macor

**Affiliations:** ^1^Experimental and Clinical Pharmacology Unit, Centro di Riferimento Oncologico (CRO) di Aviano IRCCS, Aviano, Italy; ^2^Department of Life Sciences, University of Trieste, Trieste, Italy; ^3^Department of Drug Science and Technology, University of Turin, Turin, Italy

## Abstract

The development of nanostructures for therapeutic purpose is rapidly growing, following the results obtained in vivo in animal models and in the clinical trials. Unfortunately, the potential therapeutic efficacy is not completely exploited, yet. This is mainly due to the fast clearance of the nanostructures in the body. Nanoparticles and the liver have a unique interaction because the liver represents one of the major barriers for drug delivery. This interaction becomes even more relevant and complex when the drug delivery strategies employing nanostructures are proposed for the therapy of liver diseases, such as hepatocellular carcinoma (HCC). In this case, the selective delivery of therapeutic nanoparticles to the tumor microenvironment collides with the tendency of nanostructures to be quickly eliminated by the organ. The design of a new therapeutic approach based on nanoparticles to treat HCC has to particularly take into consideration passive and active mechanisms to avoid or delay liver elimination and to specifically address cancer cells or the cancer microenvironment. This review will analyze the different aspects concerning the dual role of the liver, both as an organ carrying out a clearance activity for the nanostructures and as target for therapeutic strategies for HCC treatment.

## 1. Introduction

Nanotechnology is nowadays widely used for the disease diagnosis, delivery, and targeting of therapeutics for several types of cancers. A key role in the success of the therapeutic use of nanoparticles (NPs) is strongly played by the clearance rate occurring in the body. Published data reported that up to 99% of the NPs injected in the bloodstream are cleared, mainly by the liver. This process can affect the efficacy of NPs to effectively reach their target and exert a therapeutic effect [1], as well as potentially increase the risk of unwanted liver toxicity [2]. Biodistribution studies have demonstrated that the clearance action exerted by the liver is confirmed for the majority of NP designs: polymeric NPs [3–5], micelles [6, 7], quantum dots [8], gold NPs [9], and carbon nanotubes [10]. In this context, injected nanomaterials usually accumulate in the liver and in an amount that depends on their physiochemical properties, such as size, shape, and surface functionalization, although relatively little is understood about dynamics of NP transport at the intraorgan level [11, 12]. In the liver, clearance is also facilitated by the fact that, when NPs enter and traverse this organ, their velocity is reduced approximately 1000-fold, increasing the interaction between NPs and liver cells, subsequently favoring clearance [1].

NPs and the liver have a unique interaction in the body because the liver represents one of the major barriers for drug delivery. The interaction between NPs and the liver becomes even more relevant and complex when the drug delivery strategies employing nanostructures are proposed for the therapy of liver diseases, such as hepatocellular carcinoma (HCC). In this case, the selective delivery of therapeutic NPs to the tumor microenvironment especially collides with the tendency of nanostructures to be quickly eliminated by the liver.

HCC represents the sixth most common form of cancer worldwide. HCC usually arises from a preexistent liver disease, frequently a cirrhotic state, and is associated with well-defined risk factors including chronic viral type B and type C hepatitis, alcohol intake, and exposure to aflatoxin [13, 14]. Several therapeutic options can be applied at the early or intermediate stage of the disease including liver transplantation, resection or radiofrequency ablation (early stage), transarterial chemoembolization (TACE), or radioembolization (intermediate stage). Nevertheless, the early stages of the HCC disease are frequently asymptomatic leading to disease detection in advanced stages. In this clinical setting, the treatment with the multikinase inhibitor sorafenib is the most common treatment option [13–15]. Very recently, immunotherapy by using immune checkpoint inhibitors has been considered as a useful treatment option for HCC [16].

The design of a new therapeutic approach based on NPs to treat HCC has to particularly take into consideration passive and active mechanisms to avoid or delay liver elimination and to specifically address cancer cells or the cancer microenvironment.

This review will analyze the different aspects concerning the dual role of the liver, both as an organ carrying out a clearance activity for the nanostructures and as target for therapeutic strategies for HCC treatment.

## 2. Mechanisms Involved in the Hepatic Clearance of NPs

**Figure 1:**
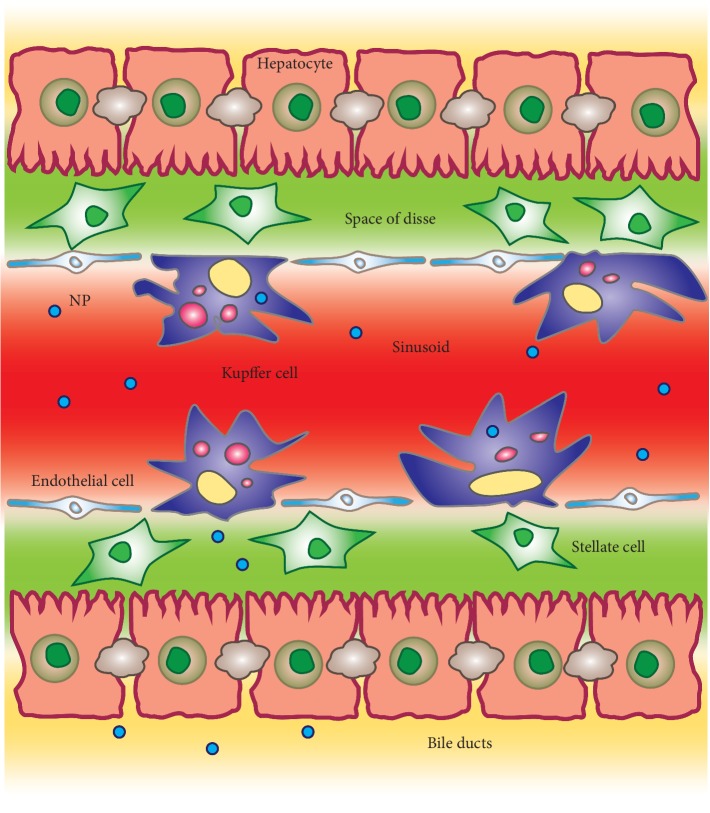
Schematic representation of the liver processing causing nanoparticles clearance.

The liver is the largest gland in humans. It is connected to two important blood vessels, the hepatic artery and the portal vein. The hepatic artery transports blood enriched of oxygen from the aorta whereas blood carried by the portal vein is enriched in digest nutrients from the gastrointestinal tract, spleen, and pancreas. Both blood vessels subdivide into liver sinusoids, small capillaries that lead to hepatic lobules. Hepatic lobules consist of plates of hepatocytes and parenchymal cells, radiating from a central vein. Each hepatic lobule is characterized by a portal triad that is constituted by five structures, a branch of hepatic artery, a branch of portal vein, a branch of the vagus nerve, a bile duct, and lymphatic vessels. Liver sinusoids are lined with two types of cells, sinusoidal endothelial cells and Kupffer cells. Hepatic stellate cells are nonparenchymal cells located in the perisinusoidal space, the space between a sinusoid and a hepatocyte (Figure 1). In the sinusoidal lumen are also often present intrahepatic lymphocytes [17]. While not apparent histologically, the sinusoid is functionally heterogeneous. Studies have shown that hepatocytes are characterized by a different gene expression throughout the sinusoid that is driven by different gradients in oxygen tension, substrate, and hormone concentrations [18–20]. The Kupffer cells are liver tissue resident macrophages characterized by highly differentiated surface receptors for the uptake of pathogens and foreign bodies passing through blood. Kupffer cells (that represent 80–90% of total body macrophages) are responsible for the most part of phagocytic activity in the liver. Kupffer cells, together with blood-circulating monocytes and splenic (red pulp and marginal zone) macrophages, constitute the mononuclear phagocyte system (MPS), which is responsible for the sequestration of more than 95% of NPs which consequently fail to reach their specific target [21, 22]. The uptake rate of NPs depends strongly on size, surface charge and ligand chemistry, and shape. It was observed that NPs, sizing several hundreds of nanometers (from 400 nm to 600 nm), are preferentially phagocyted by these cells. Also, NPs with a cationic surface are preferentially eliminated by the Kupffer cells when compared to NPs with neutral charged surface (i.e., poly(ethylene glycol)—PEG). Similarly, NPs with anionic surface could be easily eliminated since their negative surface charge can absorb circulating positively charged serum proteins forming a “protein corona,” subsequently favoring their interaction with the receptor macrophages. Also, NP shape can influence the behavior of NPs in the bloodstream and their distribution. There are no consistent data suggesting the best NP shape for a low clearance. Interestingly, studies reported that the rod-shaped NPs showed a reduced clearance when compared to the clearance rate of spherical shaped NPs; this is probably due to the presence of a few accessible binding sites available for macrophage interaction [23–25]. The resident macrophages have been observed in a 3 : 2 ratio in the periportal versus the pericentral region.

The hepatocytes in the liver endocytose NPs and can release them either back into the bloodstream or into the bile. However, this process occurs at a much lower rate compared to the Kupffer cells. Interestingly, the hepatocyte uptake was only seen when large doses of NPs are given or when macrophages are chemically depleted [26]. Once processed by hepatocytes, these NPs are cleared through bile into the feces. In particular, NPs that interact with hepatocytes can be processed and cleared from the body via the hepatobiliary system. This process starts when injected nanomaterials, circulating in the bloodstream, slow down when they enter the liver via the portal vein. This allows the nanomaterials to interact with a variety of cells, but to successfully transit through the biliary system, NPs must avoid being taken up by liver-resident Kupffer cells [27]. Depending on their physicochemical properties, first of all on their size, NPs smaller than the diameter of sinusoid fenestrations (50–150 nm) can freely diffuse into the Disse space and can be taken up by hepatocytes [17, 28]. There are also other factors that can more likely favor the uptake of NPs by hepatocytes, in particular, PEGylation and NP positive surface charge [29, 30]. Hepatobiliary clearance is generally an active process promoted by transporters and realized by many drug-metabolizing enzymes and then followed by secretion into the bile duct via bile canaliculi or either back into the bloodstream [26, 31]. However, the hepatocyte uptake occurs only with smaller size NPs (less than 50 nm) or when large doses of NPs have been injected or if macrophages have been chemically depleted [26, 32].

A minor role in NP clearance is also performed by the liver sinusoidal endothelial cells (LESCs). LESCs constitute about half of the nonparenchymal cells of the liver. Structurally, these cells separate the hepatocytes from the blood of the sinusoidal lumen, lack a basement membrane, and have fenestrate sizing from 100 nm to 150 nm, therefore allowing the passage of small NPs [33, 34].

The internalization of NPs in eukaryotic cells can be done through macropinocytosis, clathrin-mediated or caveolin-mediated endocytosis, or additional endocytic pathways, independently from size, shape, and surface charge [35]. The mechanism of LSECs internalization of NPs is performed by clathrin-mediated endocytosis [36]. Sinusoidal endothelial cells are not only the most permeable endothelial cells of the body, due to the association of fenestrae and the absence of typical basement membrane, but also one of the highest endocytic cells in the human body. This property combined with a strong lysosomal activity gives these cells the ability to remove soluble macromolecules and NPs through pinocytic receptor-ligand interaction [37]. Some major high affinity endocytosis receptors are involved, including scavenger receptors (SR-A, SR-b, and SR-H), mannose receptor, collagen-alpha receptor, and Fc gamma-receptor [17, 38]. The SRs, especially, mediate endocytosis of polyanionic molecules and also of negatively charged NPs [39, 40].

All these findings highlight that a detailed understanding of where and how NPs are sequestered and cleared within the liver is crucial to solve the targeting problem of NPs. Sinusoidal endothelial cells also differ morphologically; indeed, periportal cells show fewer and larger fenestrations in comparison with the same cells in the pericentral region, impacting the molecules that may access the underlying hepatocytes [41]. The zonal heterogeneity affects also NP distribution at the lobule level; preferential periportal accumulation was observed for gold NPs coated with PEG and quantum dots [42].

## 3. Protein Corona and Macrophage Elimination

When NPs are administered intravenously, they come in contact with a solution of thousands of proteins (62–84 g/L) that can be adsorbed on their surface conferring a new biological identity and influencing their clearance *in vivo*. The adsorption of serum proteins on NPs is termed “opsonization” and leads to the formation of a dynamic protein coat known as “protein corona” [43, 44]. The protein corona is considered to be a continuous flux of desorption/adsorption processes controlled by the so-called “Vroman effect”: at any time, an initially attached protein can desorb from the NP surface and be replaced by a different one with higher affinity. This process changes the composition of the protein corona while the amount of adsorbed proteins remains relatively constant [45–47]. The generation of the protein corona is divided into two phases: initially, more abundant proteins, with low affinity, are rapidly adsorbed on NPs, and then, they are replaced by less abundant proteins but with higher affinity, until the corona reaches a stable composition. The latter type of proteins binds irreversibly to the NP surface within seconds or a minute constituting the “hard corona”; low affinity proteins with fast exchange rates interact with the hard corona components through protein-to-protein interactions constituting the “soft corona” [45, 46, 48].

The protein corona formation is related to the physicochemical properties (i.e., size, charge, shape, and surface composition) of NPs and the amount of proteins in the biological fluid [43, 44] and can be considered unique for each nanomaterial. The protein corona contributes to NP clearance, circulation time, bioavailability, and toxicity, determining a different interaction with the cells in the body [43, 44].

The protein corona plays a fundamental role in the biodistribution of NPs, and in particular, in the recognition by the MPS, especially through the adsorption of opsonins such as immunoglobulin G (IgG), coagulation proteins (i.e., fibrinogen), or complement components, which are believed to promote recognition by specific receptors on macrophage and phagocytosis [29, 47, 49]. On the contrary, dysopsonins, such as apolipoprotein J (clusterin) and albumin, were reported to reduce the adsorption of opsonins on the surface of NPs conferring “stealth properties” [47, 50].

Current research in the field of nanomedicine is focused on modulating the protein corona formation to minimize the rapid recognition by MPS and the rapid elimination of NPs from the body. The surface of NPs can be modified with polymers that respond to the four criteria described by Whitesides and coworkers: hydrophilicity, absence of net charge and hydrogen bond donors, and presence of hydrogen bond acceptors. Among neutral polymers, PEG was considered the gold standard for protein-resistant surfaces because it corresponds to all the criteria and prevents adsorption of opsonins on NPs. However, recent studies demonstrated that the clinical use of such polymer is limited by its thermal instability, the production of toxic metabolites after its enzymatic degradation, and the formation of anti-PEG antibodies after repeated administrations of PEG or PEG conjugates [42]. In recent years, much attention has been given to alternatives to PEG, i.e., poloxamer, polyvinylpyrrolidone (PVP) and dextran [47]. Other strategies to prevent MPS engulfment of NPs include (i) the saturation of receptors expressed on Kupffer cells with nontoxic NPs prior to administration of a nanotherapeutic [51]; (ii) the specific transient depletion of macrophages by the injection of dichloromethylene-bisphosphonate- or clodronate-loaded liposomes or substances like gadolinium chloride, methyl palmitate, dextran sulfate, and carrageenan [17, 51]; (iii) bio-inspired cell-based approaches which consist in using cells, i.e., red blood cells, platelets, leukocytes, monocytes, and stem cells as drug delivery systems [52, 53].

## 4. NP Surface Modification to Improve Distribution Properties

Polymeric NPs, micelles, nanoemulsions, nanohydrogels, liposomes, and solid lipid NPs have been developed as nanodelivery systems mainly for anticancer drugs in HCC therapy exploiting passive targeting. It is worth noting that NPs after IV administration accumulate at higher levels in the liver than in other tissues, if they were not engineered for other selective targets.

Li et al. [54] described the challenge and the strategies to overcome the clinical problems of nanosystems for the treatment of liver diseases, including HCC. A number of options have been proposed; the most are related to the modification of NP surface. This strategy can include either the coating of the surface or the chemical conjugation with different molecules or targeting ligands.

Surface modifications can significantly alter the circulation lifetime by changing the surface charge, hydrophobic capability, targeting capacity, or biocompatibility of NPs [55].

The surface charge has a critical impact on the blood circulation and the biodistribution of nanocarriers, as well as on their stabilization [56]. Indeed, the surface charge contributes to the electrostatic repulsion between NPs, avoiding their aggregation, according to DLVO theory. However, the modulation of the surface charge can be considered an important balance for the system stabilization and for *in vivo* fate of NPs. Different studies investigated the effects of surface charge of different nanodelivery systems such as polymeric NPs, micelles on their in vitro cellular uptake by macrophages, cytotoxic effects, and in vivo biodistribution in xenograft models [23, 57, 58].

Xiao et al. studied the biodistribution of differently charged PEG-oligocholic acid-based micelles, showing that the liver uptake was very high for highly positively or negatively charged NPs. On the contrary, for micelles with slightly negative charge, low liver uptake and high tumor accumulation were observed [57]. Similar insights were shown in another work that compared the cellular uptake and biodistribution of negatively charged carboxymethyl chitosan-grafted NPs and positively charged chitosan hydrochloride-grafted NPs. The results suggested that NPs with slight negative charge accumulated in the tumor and in the lesser extent in the liver, while positively charged NPs showed higher liver and spleen localization [23].

NPs can be modified by coating with ionic or nonionic polymers. This approach can limit the interaction with plasma proteins and phagocytic system, prolonging the blood residence time.

The hydrophobicity of NP surface is another key factor to consider for biodistribution. Indeed, it affects opsonization, related to capture by the RES and a faster blood clearance. It was shown that the more hydrophobic the NP surface is, the higher the absorption of plasma proteins will be, after *in vivo* administration [59]. The coating with hydrophilic polymers/moieties, such as polyethylene glycol (PEG), polyethylene oxide (PEO), polyvinylpyrrolidone (PVP), polyacrylic acid, poloxamer, and poloxamine, has been proven one of the most promising approaches to avoid opsonization. Hydrophilic polymers on the surface of the nanocarrier repel other molecules by steric effects [60]. The use of PEG moieties on NP surface represents a polymer shield that can reduce the nonspecific scavenging of nanotherapeutics by RES. PEG is a neutral and hydrophilic polymer that can form a hydrophilic flexible barrier layer on the surface of NPs which produces low opsonization level, prolonged blood circulation, and the escape from the phagocytic system. For this behavior, PEG-coated nanocarriers are called stealth NPs [61]. A large number of studies highlight that the capability of PEGylation to mask the nanocarrier surface hydrophobicity/charge is largely affected on its properties, such as its length, density, and conformation [62]. Interestingly, PEG-modified PLGA NPs showed a five-fold increase in cell uptake than the unmodified NPs.

Gao and coworkers developed protamine surface-modified PLGA-*b*-PEG-*b*-PLGA NPs for the delivery of paclitaxel to the liver. These NPs were easily internalized by HCC cell line HepG2. The high cellular uptake was shown to be related to protamine modification. More recently, another paclitaxel-loaded PLGA NPs for the treatment of liver cancer were designed by Mandal et al.; prolonged half-life and higher plasma and liver drug concentrations were observed compared to the free drug in rats [63].

The modification of NPs with hydrophilic polysaccharides is an alternative to PEGylation to modify pharmacokinetic parameters of NPs [64]. Iron-oxide magnetic NPs were coated with carboxymethyl dextran or dextran, and the NP biodistribution was studied in mouse models. The coating layer minimized the interactions between the iron core and plasma proteins and enhanced the circulation time. Iron deposition from NPs possessing a negative surface potential was observed to have higher accumulation in the liver and spleen [65].

Chitosan and its derivatives have been exploited as the hydrophilic coating material for different nanoparticulated systems. Glycolchitosan-shelled nanobubbles loaded with doxorubicin showed a strong accumulation at the tumor site in a xenograft model of anaplastic thyroid cancer [66]. Interestingly, polysaccharide can interact with a particular receptor of cell membrane. This phenomenon is exploited for achieving an active targeting (i.e., hyaluronic acid).

Hyaluronic acid-modified disulfide-crosslinked PLGA-PEI NPs were developed as a target-specific paclitaxel and siRNA codelivery system [67]. These NPs modified by HA enhanced tumor targeting efficiency by the CD44 receptor-mediated uptake of NPs.

Although hydrophilic materials on the surface of NPs prevent opsonization, hydrophobic characteristics are often required to increase membrane permeability and cellular uptake. Amphiphilic copolymers or block copolymers have shown promising results addressing these issues [60].

## 5. Targeting Strategies of Drug-Loaded NPs

Nanomedicine offers unique opportunity for an active/passive targeting to convey drugs to specific target sites [68, 69]. In the last years, this technology has made remarkable improvements in NP solubility, stability, biocompatibility, and release profile of the drugs [70, 71].

The passive-targeting approach depends on the drug-loaded NPs physiochemical properties, administration route and, most importantly, on the EPR effect of the tumor vasculature [72]. Specifically, NPs can be accumulated at the tumor site due to the leaky architecture between endothelial cells and the poor lymphatic drainage. NPs can concentrate in the tumor microenvironment for a long time due to the insufficient venous and lymphatic clearance [73]. Several studies reported that different compositions of NPs, ranging from 10 nm to 200 nm, penetrated the leaky vessel walls around the tumor also due to an EPR effect [74–77]. Furthermore, significant antitumor activities, like tumor regression and long-term survival, were observed when therapeutic NPs were used. Kim et al. showed that the prolonged blood circulation of the chitosan-based NPs induced higher EPR efficiency of the NPs [78]. Yhee described that particle size, particle shape, and surface charge of NPs also affected their blood circulation time and EPR effect [79].

Although passive targeting is currently the most investigated mechanism requested in cancer nanomedicine, including HCC treatment, the rapid clearance rate of nanostructures restricts their use [80–87].

Therefore, to increase the accumulation of NPs at the tumor site, the active targeting approach is extensively being explored. In particular, this approach takes advantage of the use of a ligand which binds preferentially to tumor cells, thus increasing the accumulation rate of drug-loaded NPs in the tumor region [88, 89]. In vitro models showed that the accumulation rate of targeting tumor NPs was higher than controls [26].

In the context of HCC, specific markers are being explored for site-specific delivery of anticancer drugs. The asialoglycoprotein receptor (ASGPR) is expressed in well-differentiated forms of HCC cells. The expression of this receptor occurs in early and advanced HCC patients, and it is one of the most studied targets to selectively deliver anticancer drugs to HCC [27, 82]. This receptor has binding affinity to a long range of molecules containing galactose and *N*-acetyl-galactosamine residues such as lactose, galactoside, galactosamine, and lactobionic acid, and asialofetuin, which could be conjugated to the surface of NPs for active targeting [27]. Several studies reported an improved cytotoxic effect in HCC cells [72, 90].

Glycyrrhizin/glycyrrhetinic acid receptor is also employed in HCC drug targeting. Glycyrrhetinic acid, a pentacyclic triterpenoid, is abundantly expressed on the cellular membrane of HCC cells. Several kinds of glycyrrhetinic acid receptor-targeted polymers were used to deliver doxorubicin, 5-fluorouracil, and paclitaxel in polymeric NPs to HCC cells [91–95]. A high accumulation in HCC cells associated with tumor growth inhibition was observed [72, 96].

Transferrin receptor (TfR) is overexpressed in many malignant cells, including HCC, and has become a promising target for potential treatment [97]. TfR-modified NPs loaded with doxorubicin and cisplatin showed a higher cytotoxicity when targeting human HepG2 cells than nontargeting NPs [98, 99].

Folate receptors (FR) are glycosylphosphatidylinositol (GPI) membrane-anchored glycoproteins overexpressed in HCC compared to the normal liver. The natural ligand for this receptor is the folic acid, and its use is being explored for NP drug delivery in these cancer cells [90, 100].

Glypican-3 (GPC3), another GPI-anchored protein, is highly expressed in most liver cancers, but absent or expressed at very low levels in normal adult tissues [101–103]. The conjugation of an anti-GPC3 antibody to NPs was explored as a drug delivery system. In vitro and in vivo experiments showed that the use of anti-GPC3 polymeric NPs loaded with sorafenib inhibited HepG2 cell proliferation. Therefore, this drug delivery system could be a potential tool for the targeted treatment of liver cancer [104].

CD44 is a cell adhesion glycoprotein expressed on cellular surface and involved in immune recognition, cell-matrix interactions, and cell migration. Patients with HCC overexpress CD44, and this expression is associated with tumor growth, metastasis, and poor prognosis [105]. CD44-targeted liposomes were explored as a potential system for drug delivery targeting in HCC [106]. In vitro data reported that anti-CD44 antibody-mediated liposomal NPs loaded with doxorubicin efficiently targeted the HCC cells compared to normal liver cells and significantly reduced tumor growth [82, 107].

### 5.1. Agents for Active Targeting

One of the breakthroughs in modern medicine involves the use of highly specific molecules for antigens expressed or overexpressed on particular cell types or for the recognition of a particular pathological tissue in order to increase therapeutic efficacy with lower dosages. The ultimate goal of this rationale is to reduce the toxicity caused by off-target effects. Surely, the revolutionary discovery made by Köhler and Milstein [108] for the production of antibodies has paved the way to the so-called targeted therapy. Nowadays, there are many successful drugs based on this technology that has allowed the targeting of specific receptors, or “signature” molecules of particular tissues. However, in addition to antibodies, other targeting systems have been developed. Among these molecules, there are a large group of antibody fragments, small peptides, nucleic acids such as aptamers, vitamins, and carbohydrates [109]. This approach potentially increases therapeutic efficacy, avoiding off-target side effects. Moreover, a reduction in costs of disease management is expected because the doses required to achieve the same efficacy compared to untargeted drugs might be lower [110].

#### 5.1.1. Recombinant Antibodies and Their Fragments

From the phylogenetical point of view, the antibodies are proteins found in many different jawed vertebrates such as fish, camels, rodents, and humans [111]. The antibodies are heterodimeric proteins composed of two structural elements defined as Heavy (H) and Light (L) chains because of their aminoacidic composition and molecular weight. The heavy chain is made of 440aa with a molecular weight of 50 kDa, whereas the light counterpart consists of 220aa with a molecular weight of 25 kDa [112]. Each chain can be divided into two basic building blocks: the variable (V) domain (NH_2_-terminal), able to interact with the antigen, and the constant (C) domain (COOH-terminal), responsible for the effector functions such as macrophages binding or complement system activation. The heavy chain differs from the light chain for the number and composition of these functional domains: the light chain contains one constant domain (CL), whereas the heavy chain could be composed by either three or four constant domains (CH). A spacer hinge region is located between CH1 and CH2 and confers particular flexibility to the final structure [113]. Depending on the constant composition, the human heavy chains could be classified into 5 isotypes: IgM, IgG, IgA, IgD, and IgE. The classes IgM and IgE have four CHs in contrast with the others, which have only three constant domains and the hinge region. In the variable domain, three hypervariable loops are responsible for the antigen identification, called complementarity determining region (CDR) [114].

Antibody functional domains can be identified by enzymatic digestion: two identical Fab, “fragment antigen-binding,” which consist of the whole L-chain linked with the VH and the CH1 portion of the heavy chain can be produced using papain. Instead, the immunoglobulin can be cleaved by pepsin producing F(ab′)2 fragment and a Fc fragment. The Fab can be separated into a variable fragment (Fv), made of VH and VL and responsible for the antigen binding.

Taking advantage of these functional domains, different targeting molecules have been designed and produced by recombinant technology. Improvement of the molecular biology technique allows researchers to reduce the murine component of monoclonal antibodies in favor of the human component, generating different kinds of antibodies such as chimeric, humanized, or fully human. The most successful engineered antibodies used are the scFv (single-chain fragment variable) or Fab. However, in order to improve their monovalent binding, diabodies have been produced; they consist of two scFvs fused together through a linker. Some issues are associated with their small molecular size: fast clearance and poor stability. In order to solve these problems, two different engineered antibodies have been designed: the scFv-Fc, made of the scFv fused to the CH3-CH2 portion, and the so-called “minibody,” made of the scFv fused to the only CH3. These formats showed improved pharmacokinetics, with a prolonged circulation time, increased tissue penetration, and enhanced epitope selectivity [115]. Then, the scFv-Fc has been implemented with homing peptides fused at the end of the CH3, giving this molecule a double binding capacity. It is the case of an anti-C5 fused to a synovial tissue homing peptide, capable to block the C5 of the complement system specifically into the inflamed synovial tissue [116, 117]. Using the same strategy, the same scFv-Fc has been fused to the RGD peptide in order to specifically block the complement system onto the endothelium of inflamed kidneys [118]. Following the rationale to produce an antibody with a double specificity, bispecific antibodies have been generated. These kinds of antibodies are made of two different monomers, which, due to a knob into hole mechanism, are able to self-assemble in a scFv-Fc antibody [119]. According to this strategy, a bispecific antibody can be generated, one arm of the molecule with an antigen tissue specificity and the other one able to block an inflammatory cytokine [3] or a protein of the complement system [120].

#### 5.1.2. scFv to Target Cancer

Several scFvs have been identified and tested in preclinical studies to target and treat many different pathologies encompassing autoimmune [121], neurological [122], and oncological diseases [123]. A number of studies have been carried out to isolate scFvs capable of specifically targeting signature proteins of a particular cancer cell, mainly exploiting the phage display technology [124]. Many scFvs have been generated against known proteins overexpressed by a number of different kinds of cancer cells, such as HER [125] and EpCAM [126], or against target proteins that are essential for cancer growth and spreading, such as VEGF [127] (involved in neo-angiogenesis). Recently, a novel scFv able to bind CD24, a target overexpressed by hepatocellular carcinoma (HCC), has been developed, showing a higher accumulation in the hepatocellular carcinoma xenograft mouse model and proving its potential as a therapeutic and diagnostic agent [128]. Another promising target expressed by malignant hepatocytes called Glypican-3 (GPC3) has already been exploited to isolate different scFvs with future potential applications [129, 130].

#### 5.1.3. Peptides as Targeting Agents

Although antibodies are used as targeting agents able to delivery nanocarriers in a specific tissue, recently peptides have been used with great success. Due to their small size, high stability, low immunogenicity, and easy production, peptides have huge potential as targeting agents [110, 131]. Peptides containing the aminoacidic sequence Arg-Gly-Asp (RGD) are able to bind integrins α_v_β_3,_ which are expressed on endothelial cells of the inflamed tissue and tumors. Indeed, in a mice model of a syngeneic transplantable liver tumor, a retarded tumor growth and a prolonged survival time of mice treated with paclitaxel- (PTX-) loaded RGD-NPs have been demonstrated when compared to nontargeted NPs [132]. In the same way, it has been proved that RGD-MTX-PLGA-Au NPs injected into a model of collagen-induced arthritis had a superior therapeutic efficacy, compared with untargeted NPs, with a much smaller dosage of MTX in the NPs [133]. More recently, Gao et al. tested RGD-coated nanodots as the theranostic agent for HCC, both in vitro and in vivo [134]. Another well-known peptide used to target endothelial cells of cancer blood vessels is the Asn-Gly-Arg (NGR). It binds the CD13 expressed on tumor endothelial cells and in a clinical trial, the NGR, conjugated with the human tumor necrosis factor (NGR-hTNF), showed no toxic effects and a progression-free survival (PFS) rate of 2.3 months compared with the control group [135]. Other peptides have been isolated to target chronic inflammatory diseases like rheumatoid arthritis. Indeed, Lee et al. have isolated a peptide with specificity for the human arthritic synovium, in particular, for the inflamed microvasculature, by injecting a phage display library in the human synovium-SCID mouse transplantation model [136, 137]. This peptide allows a selective delivery of polymeric NPs in synovial tissue and an effective therapeutic control of the inflammatory process [138].

**Figure 2:**
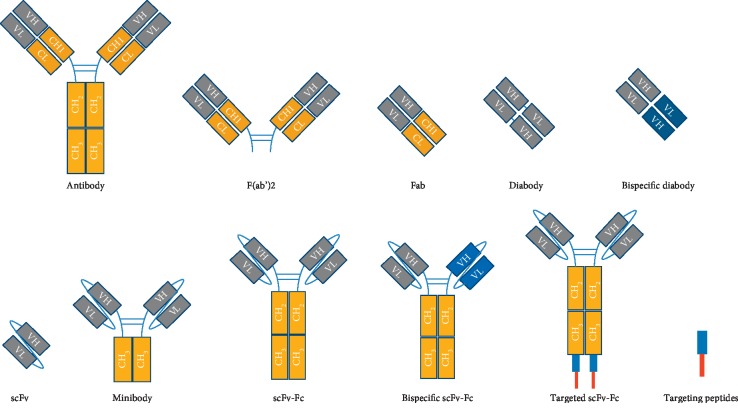
Schematic representation of potential agent for active targeting of nanostructures.

Figure 2 summarizes the structure of a full immunoglobulin with the VL, VH, CL, and CH. Then, fragments such as F(ab)_2_ and Fab' and other formats like scFv and scFv-Fc are reported. Finally, there are represented by small targeting molecules such as peptides and aptamers (figures adapted from Reference [109]).

## 6. Clinical Trials Investigating the Use of NP-Based Therapy for HCC Treatment

To date, there are several available therapies in clinical use (like resection or radio frequency ablation (RFA), transarterial chemoembolization (TACE) or radioembolization, and chemotherapy) [139]. However, it still remains a treatment challenge to overcome the actual poor survival rate, in particular, regarding the advanced-staged hepatocellular carcinoma (HCC) patients (<5%) [140].

Many NP formulations of anticancer drugs are approved for human use and are already available in the market [141–143]. In the HCC field, there is only one nanodrug clinically approved in Europe and Asia, the doxorubicin-eluting beads trans-arterial chemoembolization (TACE). Compared to the conventional TACE, this technology exerts both the therapeutic components of TACE, that is, the drug-carrier function and embolization, thus minimizing the risk of systemic drug [144]. The beads are composed of a hydrophilic, ionic polymer that can bind and load the drug (i.e., doxorubicin) positively charged. After its administration in liver tumor by intra-arterial injection, these microspheres begin to slowly release the doxorubicin in a controlled manner [145–147]. However, despite promising results of doxorubicin DEB-TACE, novel nanotechnologies are currently being investigated in clinical trials (Table 1).

Currently, there are 3 clinical trials for HCC treatment in phase III involving nanostructures, whose endpoint is to investigate the antitumoral efficacy.

The OPTIMA clinical trial (NCT02112656) aimed to determine the Thermodox technology efficacy in the treatment of non-resettable HCC. This novel nanomedicine composition is based on liposomes which encapsulate doxorubicin. When heated by the application of RFA (to 39–42°C), the heat-sensitive liposomes change their structure creating openings that release doxorubicin directly into and around the targeted tumor (https://clinicaltrials.gov/ct2/show/study/NCT02112656?term=OPTIMA+celsion&rank=1) [80, 148]. This study is still ongoing (http://investor.celsion.com/news-releases/news-release-details/celsion-announces-enrollment-completion-pivotal-phase-iii-optima).

The ADI-PEG 20 clinical trial (NCT01287585) is based on the use of an arginine deiminase (ADI) enzyme conjugated with polyethylene glycol (PEG) to treat patients with advanced HCC who failed prior systemic therapy [81]. The role of this enzyme is to deplete the circulating arginine necessary for the HCC cell proliferation. The study reported that the administration of ADI PEG 20 was safe, well tolerated by patients but did not significantly improve overall survival (https://clinicaltrials.gov/ct2/show/NCT01287585?term=NCT01287585&rank=1) [82]. However, a trend of improved survival was observed in the subgroup of patients with low levels of circulating arginine (<10 *µ*M). Therefore, new studies will focus on the potential effect of this aspect [149].

The Livatag study (NCT01655693) investigated whether Doxorubicin Transdrug (DT) is effective in the treatment of patients affected by advanced HCC after failure or intolerance to Sorafenib treatment. DT is a NP formulation of doxorubicin obtained with water insoluble poly(iso-hexyl-cyanoacrylate) polymer. This formulation easily delivers the drug into the tumor cell increasing the drug payload in the DNA target, thereby bypassing the mechanisms of multidrug resistance developed by tumor cells (https://clinicaltrials.gov/ct2/show/NCT01655693?term=NCT01655693&rank=1; https://www.onxeo.com/onxeo-announces-top-line-results-relive-phase-iii-study-livatag-advanced-hepatocellular-carcinoma/) [82].

The current phase I/II clinical trials for HCC treatment involving nanostructures, whose final endpoint was the evaluation of the nanodrug toxicity and safety, are listed as follows.

The TKM-080301 study (Arbutus Biopharma, NCT02191878) is based on the use of TKM-080301 that is composed of a short-interference RNA (siRNA) within a lipid particle, capable to be accumulated within tumors, taking advantage of the “enhanced permeation and retention (EPR)” effect [83, 150]. The siRNA target is the polo-like kinase 1 (PLK-1) protein which is overexpressed in HCC cells (up to 12-fold higher compared to controls) and promotes cell proliferation. In vitro data showed that inhibition of PLK1 activity in proliferating cancer cells rapidly induces mitotic arrest and apoptosis [151] and increases the sensitivity of cancer cells to the cytotoxic effects of chemotherapy [84].

The lipid components of TKM-080301 also protect the siRNA from degradation by plasma and tissue nucleases, prevent rapid clearance of the siRNA, and enable effective intracellular uptake of the PLK1-targeting siRNA into cancer cells [152]. The study was declared to be concluded in February 2019, and preliminary data results showed that this treatment was safe and well tolerated. The next endpoint of the study is to evaluate the antitumor activity of TKM-08030120.

A second study is the NBTXR3 study (Crystalline NPs and Stereotactic Body Radiation Therapy in the Treatment of Liver Cancers, NCT02721056). This study aimed to evaluate the safety and tolerability of NBTXR3, NPs administered by intralesional (IL) or intra-arterial (IA) injection and activated by Stereotactic Body Radiation Therapy, in the treatment of HCC. NBXTR3 is constituted by hafnium oxide NPs developed to increase the tumor-localized high energy deposit once activated by ionizing radiations such as stereotactic body radiotherapy (SBRT) and thus increasing tumor cell death compared to the same dose of radiation. From preliminary data, NBTXR3 showed no toxicity and was well tolerated. Evaluated in other tumors, NBTXR3 was successful in a phase II/III trial in soft tissue sarcoma (NCT02379845) and is currently being evaluated in head and neck (NCT01946867; NCT02901483), prostate (NCT02805894) and rectum cancers (https://clinicaltrials.gov/ct2/show/NCT02721056?term=NCT02721056&rank) (http://ascopubs.org/doi/abs/10.1200/JCO.2018.36.15_suppl.e16194) [153].

The DCR-MYC study (NCT02314052, Dose Escalation Study of DCR-MYC in patients with Hepatocellular Carcinoma) was designed for solid tumors including HCC. This nanodrug is a synthetic double-stranded RNA in a stable lipid particle suspension that targets the oncogene c-myc. The oncogene c-myc is abnormally expressed in tumors and implicated in promoting cancers [85, 142]. DCR-MYC is a Dicer substrate small interfering RNA (DsiRNA), a double-stranded synthetic RNA within a stable lipid particle, which specifically targets the oncogene c-myc (https://clinicaltrials.gov/ct2/show/results/NCT02314052?view=results).

MRX34 (mirna Therapeutics, PhI, NCT01829971) is a synthetic double-stranded RNA which mimics microRNA-34a (miR-34a), a miRNA that downregulates the expression of >30 oncogenes across multiple oncogenic pathways, but it is lost or underexpressed in many malignancies. Contradictory information was reported on a number of occasions, especially with the use of a miRNA-34a inhibitor for HCC, making it difficult to evaluate a clear clinical benefit of this potential therapy [86].

The MTL-CEBPA study in patients with advanced liver cancer (NCT02716012) evaluated the safety and tolerability of a “short” activating RNA (saRNA) in HCC patients [87]. MTL-CEBPA comprises a double-stranded RNA inside liposomal NPs which specifically target the CEBPA gene. Downregulation of the CEBPA gene showed to inhibit the HCC tumor growth in preclinical models [154]. MTL-CEBPA is the first saRNA and the first drug targeting C/EBP-*α* to enter clinical trials. To date, the study is still ongoing (https://clinicaltrials.gov/ct2/show/NCT02716012?term=NCT02716012&rank=1; http://ascopubs.org/doi/10.1200/JCO.2017.35.15_suppl.TPS2612).

## 7. Conclusions

The development of nanostructures for therapeutic purpose is rapidly growing, following the results obtained in vivo in animal models and in the clinical trials. Information collected up to now confirmed the safety of this therapeutic approach. Unfortunately, the potential therapeutic efficacy is not completely exploited, yet. This is mainly due to the fast clearance of the nanostructures in the body and liver was immediately shown as the organ deputized to a fast elimination. Physicochemical modification of NP surface, as well as changes in their shape, provide the possibility to increase the half-life of the nanostructures and enhance their possibility to take advantage of the EPR effect in cancer or inflammatory microenvironment.

The application of nanomedicine in liver diseases, and in particular in HCC, represents the most evident example of the limits in the application of these therapeutic approaches. The leaky vessels of the liver have similar characteristics of the leaky vessels in cancer; the presence of macrophages, endothelial cells and hepatocytes tends to phagocytes nanostructures; the interaction of the particles with plasma proteins enhance their fast elimination. The result is a very low accumulation of the nanosystems in the pathological part of the liver. In the on-going clinical trials, the treatments of HCC using NPs for the delivery of different drugs demonstrated their safety but exploit only EPR effect in contrast with the elimination effect caused by the healthy structures of the liver.

**Figure 3:**
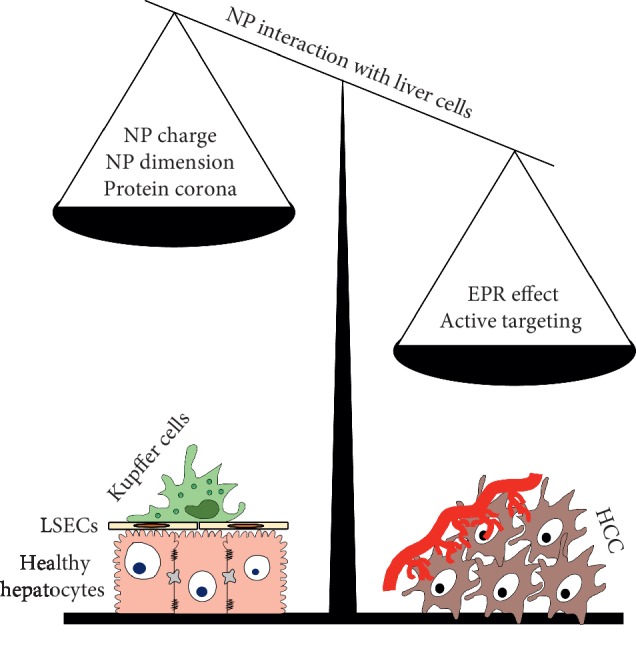
Schematic representation of the factor influencing selective delivery of nanoparticles developed for HCC treatment.

A possible turning point could be the use of targeting agents covalently bind of particle surface; this approach demonstrated a superior therapeutic effect in different animal models. Its application depends from the individuation of specific tumor-associated antigens and from the development of molecules avidly able to bind them. Recent studies have individuated several molecules that can represent possible targets for the selective delivery of targeted NPs. This probably represents the next step in the nanomedicine for the treatment of HCC, as summarized in Figure 3.

## Figures and Tables

**Table 1:** The reported clinical trials investigating the use of nanostructures whose endpoint is the treatment of hepatocellular carcinoma.

Clinical trial name	Phase	NP type	NP target	Trial number
OPTIMA	III	Heat-sensitive liposome, doxorubicin loaded	Non-active targeting	NCT02112656
ADI-PEG 20	III	Polyethylene glycol (PEG) conjugated with arginine deaminase (ADI) enzyme	Depletion of arginine	NCT01287585
Livatag study	III	Formulation of doxorubicin with water insoluble poly(iso-hexyl-cyanoacrylate) polymer	Non active targeting	NCT01655693
TKM-080301 study	I/II	Short-interference RNA (siRNA) within a lipid particle	Downregulation of polo-like kinase 1 (PLK-1) protein	NCT02191878
NBTXR3 study	II/III	Hafnium oxide NPs developed to increase the tumor-localized high energy deposit once activated by ionizing radiation such as stereotactic body radiotherapy (SBRT) and thus increasing tumor cell death compared to the same dose of radiation	Non-active targeting	NCT02379845; NCT01946867; NCT02901483.
DCR-MYC study	I	Double-stranded RNA in a stable lipid particle suspension	Downregulation of oncogene c-myc	NCT02314052
MRX34 study	I	Double stranded RNA which mimics microRNA-34a (miR-34a) within liposomal NPs	Downregulation of miR-34a targets	NCT01829971
MTL-CEBPA study	I	Short activating RNAs (saRNA) within liposomal NPs	Downregulation of CCAAT enhancer binding protein alpha (CEBPA) gene	NCT02716012

NCT, number of clinical trials.
